# Editorial for the Special Issue on Uncertainty in the Brain

**DOI:** 10.1186/2190-8567-4-7

**Published:** 2014-04-17

**Authors:** Olivier Faugeras, Michèle Thieullen

**Affiliations:** 1 NeuroMathComp team (INRIA), Sophia Antipolis, France; 2 INRIA, 2004 Route des Lucioles, 06902, Sophia Antipolis, France; 3 Laboratoire de Probabilités et Modèles Aléatoires, UMR 7599, Université Pierre et Marie Curie—Paris 6, Boîte l88, 4, Place Jussieu Cedex 05, 75252, Paris Cedex 05, France

## 

### 

Theoretical neuroscientists have developed a wide range of mathematical, computational, and numerical tools for modeling and simulating sets of interacting neurons. While in most cases, with some notable exceptions, the framework of these efforts has been deterministic, drawing on the theory of dynamical systems, partial differential equations, integral or integro-differential equations, it has been felt from the early days of Hodgkin and Huxley that uncertainty has to be taken into account in the models. Uncertainty has its source in the physics of the underlying phenomena, for example in the way the ion channels open and close, or the way in which neurotransmitters diffuse in the synaptic cleft. This is the physical uncertainty. Uncertainty also comes from the fact that many of the parameters in the models are out of reach of any of the current experimental techniques, and most likely will still be for a long time. For example the exact values of the synaptic weights describing the way neurons influence each other at a given instant in a network will probably never be known. This second source is the intrinsic uncertainty. Researchers are therefore in great need of stochastic models to account for these two sources in a mathematically rigorous framework allowing for quantitative descriptions and predictions.

### 

The papers that appear in this special issue are successful attempts in the direction of building up, analyzing, and testing such models on real data.

### 

The one by Deena Schmidt and Peter Thomas addresses the problem of finding the optimal complexity reducing mapping from a stochastic process on a graph to an approximate process on a smaller sample space, as determined by the choice of a particular linear measurement functional on the graph. They use recent results from random matrix theory to provide heuristic error estimates for the accuracy of the stochastic shielding approximation for an ensemble of random graphs. They then demonstrate the use of their theory for the partitioning of ion channel states into conducting versus nonconducting states.

### 

The paper by Davide Barbieri, Giovanna Citti, and Alessandro Sarti focuses on the mathematical analysis of the shape index of simple cells. Their analysis is based on the Uncertainty Principle and provides quantitative bounds on the family of simple cells that are found in primary visual cortex.

### 

The paper by Iolov, Ditlevsen, and Longtin focuses on the analysis of sinusoidal noisy leaky integrate-and-fire models. These authors propose two methods to estimate input parameters using interspike interval data only. One is based on numerical solutions of the Fokker–Planck equation, and the other is based on an integral equation, which is fulfilled by the interspike interval probability density. The methods are compared on simulated data.

### 

The paper by Patricia Reynaud-Bouret, Vincent Rivoirard, Franck Grammont, and Christine Tuleau-Malot deals with the statistical analysis of spike trains. These authors propose a new method based on subsampling to deal with the plug-in issues in the case of the Kolmogorov–Smirnov test of uniformity. They show the performance of their method on simulated data and provide a complete analysis on single unit activity recorded on a monkey during a sensory–motor task.

### 

The paper by Grégory Dumont, Jacques Henry, and Carmen Oana Tarniceriu is centered on the analysis of population density model arising from the theta-neuron model. They prove the existence and uniqueness of a solution to the population model and present some numerical simulations.

### 

The paper by Christian Kühn and Martin Riedler focuses on the effect of additive noise on integro-differential neural field equations. They demonstrate that an efficient finite-dimensional approximation of the stochastic neural field equation can be achieved using a Galerkin method and that the resulting finite-dimensional rate function for the LDP can have a multi-scale structure in certain cases. Their approach also provides the technical basis for further rigorous study of noise-induced transitions in neural fields based on Galerkin approximations.

### 

Although mathematical work on probabilistic and statistical approaches to neural dynamics has increased in recent years, our understanding of the role of uncertainty in shaping up the brain activity is still in its infancy. We look forward to the time when more mathematicians will take up this grand challenge and use the Journal of Mathematical Neuroscience as a forum for the exchange of results and ideas.

## Authors’ Contributions

Both authors contributed equally to the writing of this paper.

